# Managing Transition with Support: Experiences of Transition from Child and Adolescent Psychiatry to General Adult Psychiatry Narrated by Young Adults and Relatives

**DOI:** 10.1155/2014/457160

**Published:** 2014-01-20

**Authors:** Eva Lindgren, Siv Söderberg, Lisa Skär

**Affiliations:** Department of Health Science, Division of Nursing, Luleå University of Technology, SE-97187 Luleå, Sweden

## Abstract

Young adults with mental illness who need continuing care when they turn 18 are referred from child and adolescent psychiatry to general adult psychiatry. During this process, young adults are undergoing multiple transitions as they come of age while they transfer to another unit in healthcare. The aim of this study was to explore expectations and experiences of transition from child and adolescent psychiatry to general adult psychiatry as narrated by young adults and relatives. Individual interviews were conducted with three young adults and six relatives and analysed according to grounded theory. The analysis resulted in a core category: managing transition with support, and three categories: being of age but not mature, walking out of security and into uncertainty, and feeling omitted and handling concerns. The young adults' and relatives' main concerns were that they might be left out and feel uncertainty about the new situation during the transition process. To facilitate the transition process, individual care planning is needed. It is essential that young adults and relatives are participating in the process to be prepared for the changes and achieve a successful transition. Knowledge about the simultaneous processes seems to be an important issue for facilitating transition.

## 1. Introduction

Young adults with mental illness who need continuing care when they turn 18 are referred from child and adolescent psychiatry (CAP) to general adult psychiatry (GenP). During this process, young adults are undergoing multiple transitions as they come of age while they transfer to another unit in healthcare [1]. During the transition from childhood to adulthood, they have to adjust to more independent living [2], incorporate new knowledge, and begin to regard themselves as adults [3]. This transition can be especially critical for young adults with mental illness [4, 5], since they can be less prepared than their peers to take responsibility for themselves [6]. Therefore, there is a need for a holistic view of transition wherein both developmental and situational aspects are taken into account [3, 7].

According to Paul et al. [8], there are differences between transfer and transition. Transfer implies the event of closure of care at CAP and reestablishment of care at GenP, while transition is the process requiring therapeutic intents. Criteria for optimal transition are stated as continuity of care, a period of parallel care or joint working, at least one transition planning meeting, and handover of information. Research showed that transfer is common but successful transition is rare [8] and there is a salient risk for disruption in continuity of care for young adults during transition from CAP to GenP [9].

Rigid boundaries between disciplines can be a disadvantage [10] and the transition process can be affected when different care cultures and different perspectives meet, that is, family-oriented care at CAP and individual-oriented care at GenP [11]. Differences in approach according to diagnosis may also create barriers in transition [4, 12, 13], as well as professionals' lack of knowledge about each others' workplaces, lack of a mutual understanding, and absence of cooperation [11]. During the transition, young adults need to make a decision whether their relatives should be involved in care, and a lack of support from relatives can make young adults vulnerable. Transition can be facilitated through cooperation and transition planning [14, 15], good relationships with professionals, and support from relatives [15].

About half of all lifetime cases of mental illness start by the age of 14, and therefore prevention and early treatment need to focus more on young adults [16]. Engqvist and Rydelius [17] showed that around one-third of young adults at CAP need care from GenP in adulthood and Ramirez et al. [18] showed that around one-fourth of patients at GenP aged 18–25 had previously received care at CAP. However, there is to our knowledge a lack of studies about young adults with mental illness transitioning from CAP to GenP, especially research describing the young adults' experiences of transition. In Sweden, CAP and GenP can either be organized together in the same organization or separated [19]. In the northern part of Sweden, where this study was performed, these two disciplines are organized separately, with different heads of organizations and different economics. To be able to facilitate a transition in line with the needs of the young adults who are transferred from CAP to GenP, it is important to account for their experiences of the transition process. Thus the aim of this study was to explore expectations and experiences of transition from CAP to GenP as narrated by young adults and relatives.

## 2. Method

Grounded theory (GT) described by Corbin and Strauss [20] was selected as a suitable method for reaching the aim of this qualitative study. By using this version of GT, experience could be explained in context and processes and sequences of action could be described.

### 2.1. Settings and Participants

Consistent with grounded theory methodology, a theoretical sampling [20] was applied to gain a deeper understanding and facilitate the development of the conceptual framework that were the focus of this research. The study was performed in two outpatient units of CAP located in the northern part of Sweden. The participants were recruited when it was time for terminate care at CAP and referral to GenP. They were invited to participate by their therapist at CAP, who gave them a letter with information about the study, and obtained informed consent. The young adults' experience of transition was narrated by the young adults and relatives separately. The total number included nine participants, three young adults (two girls, one boy) and six relatives (four mothers, one father, one keyworker at a treatment home). The regional Ethical Review Board approved the study.

### 2.2. Data Collection and Analysis

Data was collected with individual interviews at the time when all young adults had ended their relations at CAP and some had their first meeting at GenP. The interviews took place in the participants' homes, at the university, or in a room at the CAP unit. The initial questions were the same for all interviews and were as follows: “could you please tell me about why you first had contact with CAP?” and “how long has the contact lasted?” Follow-up questions were asked during the interview concerning “how the participants had been prepared for transfer,” “how they were informed,” “closure of relations in CAP,” the “beginning of new relations in GenP,” and “expectations and experiences of the transition.” The data collection was carried out in parallel with studies of literature and data analysis in order to stimulate a theoretical sensitivity, that is, make the sampling open and flexible to contribute ideas in accordance with the approach in grounded theory [20]. Concepts that were derived from data during the analysis and questions about those concepts affected the next interview in data collection. This resulted in an interview guide that had new questions added verbatim, for example, how relatives experienced giving support and what young adult expectations were of their coming of age. Each interview lasted between 30 and 80 minutes (md = 50) and was digitally recorded, transcribed, and analysed verbatim.

The analysis started directly after the first interview was performed by reading through the interview text to obtain a feeling for the participant's experiences of transition. The overall analysis process was performed by open coding line by line and activity by activity, followed by axial coding and integration [20]. Open coding was the initial step of the analysis that pertains to defining concepts in order to discover categories and their properties and dimensions. The first author performed the coding process through breaking the data apart and asking questions such as “what,” “why,” “when,” and “with what consequences” and made notes that also sought activities in the data. By using constant comparison [20], different pieces of data were compared for similarities and differences. Data were coded and similar codes were then grouped into categories (Table 1). Codes like “feeling secure,” “trustful relationship,” and “feeling seen and confirmed” were grouped together into the subcategory “leaving secure relations behind.” The Internet program Open Code 3.6 [21] was used during this coding process. It is a program for coding data in qualitative studies and was developed to follow the first steps in GT.

The analysis process also included an axial coding where the act was to relate categories to one another by specifying properties and dimensions of higher-level concepts. In reality, the different steps undertaken during the data analysis were not linear. Instead, analysis was conducted through the constant comparing of data, emerging codes, and categories [20]. Based on the analysis, six subcategories were grouped together into three categories. Finally a core category was defined by an integration of all categories and concepts. In the core category all categories are related and linked together.

## 3. Results

The results are presented in a core category, managing transition with support, and the three categories, are being of age but not mature, walking out on security and into uncertainty, and feeling omitted and handling concerns. The core category and categories are presented below with quotations from the interview texts.

### 3.1. Core Category: Managing Transition with Support

**Figure 1:**
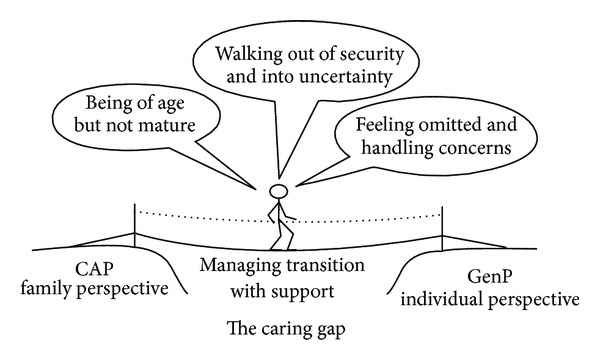
Managing transition with support. A description of how young adults and their relatives need support to manage the transition and avoid the caring gap.

The transition from CAP to GenP is a time-consuming process without clear structure, except for the time when young adults reach the age of 18 and no longer have the benefit of child and adolescent health services. The core category describes how young adults and their relatives need support to manage the transition and avoid the caring gap and that their main concerns were that they might be left out and feel uncertainty about the new situation. The interpretation of the transition process from CAP to GenP showed that young adults may not be mature enough to take responsibility even though they come of age. The young adults needed support during the transition process when they had to leave secure relations behind, create closure, and start again. It was also essential for the relatives to get support so they could be close to the young adults while also letting go. During the transition process the young adults expressed that they felt left to their fate with the risk of falling into the caring gap, which in turn could lead to an insecurity that needed to be relieved (Figure 1).

### 3.2. Being of Age but Not Mature

This category is constructed from two subcategories: still in need of support and being close yet letting go.

#### 3.2.1. Still in Need of Support

When young adults transfer to GenP, the requirements to manage themselves increase as they have to make their own decisions about continuity of care. At CAP, professionals were forthcoming with, for example, dates and reminders of appointments, but the young adults had no expectations that professionals at GenP will give support and reinforcement in the same ways they experienced at CAP. Both young adults and relatives realise that the different care cultures could be a hindrance for the young adults to accomplish treatment, especially in case of a lack of support from relatives.

*I think they are more obliging at CAP, you get times for meetings and a phone number to call, but at GenP you have to contact them and get in touch and check that everything is working…that's the big difference…it's like written that you can fix it by yourself. (young adult)*

Relatives described that it was not easy for young adults to grow up and it was not certain that the young adults became mature only because they turned 18 and come of age. It could be a struggle between desires to fend for themselves and the frightening prospect of taking responsibility. It was not obvious that young adults look forward to their 18, birthday because they know it entails obligations that may be difficult to meet. Relatives expressed that the young adults need awareness of the value of continuing care and the ability to be responsible for their treatment.

*…need [of support], yes she is in great need but does not want it…but she has to mature and realize that. (relative)*

Young adults and relatives suggested that the transition should be planned with regard to personal needs and maturity and according to possibilities of receiving support during the process. They also suggested that professionals should take into account to what extent the young adults were in need of care after transfer and provide the ability to terminate treatment at CAP if only a few appointments at GenP are needed.

#### 3.2.2. Being Close Yet Letting Go

To undergo transition and become an adult was described as a step into adulthood and independence. Relatives noted that this transition might be easy for young adults who were prepared and mature and had support from relatives as a backup when needed. Relatives further described that it could be hard to know what kind of support the child may need, but they understood they cannot be involved in decisions as they were before. To what level relatives will be able to participate and give support depends on the relationship between the young adult and the relative. Relatives expressed frustration about being close but not having the possibility of participating in the planning of care.

*I think it's horrible…not because I mistrust GenP but because I feel that I do not have control…she does not want us to be involved. (relative)*

Some relatives said that their young adults were in need of support from them to manage daily life and that it was difficult to let them go. They also felt worried about their young adults' futures and how they would manage to find a job and a place in society. One relative described that she needed to be one step ahead to support the young adult, and it became important for the relative to receive sufficient information during the transition process. Relatives described further that participating during the transition could consist of support with structure, without involvement in treatment. They expressed that independence cannot be forced but must take place gradually.

*I hope she leaves home in the long run…I want her to be at home yet for a while…say that she first got a job and then managed the medication by herself, and then in the long run…it must work out, you have to try. (relative)*

### 3.3. Walking Out of Security and into Uncertainty

This category is constructed from two subcategories: achieving closure and starting again and leaving secure relations behind.

#### 3.3.1. Achieving Closure and Starting Again

The young adults and the relatives described that they tried to negotiate terms of the transfer but were denied by the system. The decision to transfer the young adult to GenP was governed by rules that had to be followed. Participants appreciated when professionals broke the rules and let the young adult stay at CAP for some extra months.

*I do not know, I did not want it at all, but I suppose it was the best way…they had no choice…that's laws and regulations that says so and then it is simply to obey. (young adult)*

The experiences of how the transition process was carried out varied. Some young adults and relatives got a letter or a phone call with information about an appointment at GenP, and some experienced a well-planned process with an extensive involvement from the young adult, the relatives, and professionals from both CAP and GenP. The young adults and the relatives desired that the professionals from GenP come to CAP, where the family felt secure, and provide information about GenP to facilitate the process and give the young adult an opportunity to establish the new relationship before closure at CAP.

*It's GenP that should come to CAP and tell what it's like…they forget that, they do the opposite…it's not CAP that should tell us about the other…because that's when you feel you're welcome, now you feel like you've been rejected. (relative)*

To further facilitate the transition process, the young adults and relatives expressed that professionals at CAP should initiate the cooperation because they have knowledge about the family and the current needs to be met. The process should be planned according to individual needs despite age limit. Young adults and relatives expressed the importance of having enough time to establish new relationships and also gain sufficient information, providing both to the young adults and the relatives.

*What information did you get about GenP? None, it feels like I'm walking into darkness. (relatives)*

These factors were described as essential to reach a successful transition. Relatives also explained that if transition planning started before the young adults' 18th birthdays, the relatives' possibility to get involved in the process increased.

#### 3.3.2. Leaving Secure Relations behind

Young adults described long-lasting relationships and continuity among professionals as one reason that they felt safe at CAP. They appreciated being able to meet the same professionals at each visit at CAP and sometimes the same professionals over several years of care. They had also met the same physician, which eliminated the need to repeat their story over and over again. Relatives expressed that professionals with competence and long experience contributed to a feeling of being secure at CAP. Both young adults and relatives expressed the desire to not have to terminate the caring relations, as the young adults wanted to avoid changing relationships and developing new ones.

*We are secure with them and they always have knowledge about our problems. She thought it was good to know that they know. (relative)*

Being treated with respect and taken seriously and having the possibility to be involved in decisions contributed to a feeling of security, according to the young adults and relatives. Relatives described that they easily could blame themselves for their child's mental illness and pointed out how important support and encouragement from professionals was to manage that feeling of burden. Supportive relations led to a care environment at CAP that felt safe and secure.

*It's more the people, and so the environment that makes it much different, it's a more cheerful environment, it feels like, what to say…it's a better environment to grow in. (young adult)*

Both young adults and relatives described that it takes time to create a trusting relationship, and they were concerned about how to manage that after the transfer to GenP. Preconditions for a successful relationship were continuity as well as feeling confident that the professionals had skills to give treatment based on needs. Overall, the young adults had more positive expectations and fewer worries than the relatives, which helped them to believe in the future and to believe that the transition should be successful. A young adult with good experiences talked about what she was expecting from the new therapist.

*I will get along well with her, she'll understand me well and I shall thus be able to understand myself without me having to, like explain myself all the time. (young adult)*

The young adults and the relatives also described negative expectations and worries about the transition and were not convinced that care would function properly after the transfer to GenP. The future felt uncertain due to a lack of sufficient information about GenP.

### 3.4. Feeling Omitted and Handling Concerns

This category is constructed from two subcategories: left to their fate and insecurity that needs to be relieved.

#### 3.4.1. Left to Their Fate

During the transition process young adults and relatives felt omitted due to disruption in the continuity of care. During the waiting time between ending at CAP and starting at GenP the young adults described that they had to be strong by themselves or rely on support from relatives. They could handle the situation because of previous knowledge about GenP and because of personal qualities.

*You should probably not be that ill, you get to struggle a bit yourself too, but if you are too ill, it is hard to do that. (relative)*

Young adults said that the differences in the care cultures between CAP and GenP also gave a feeling of being omitted and feelings of uncertainty. A young adult described that at CAP, there was always continuity in care, and the experience from the first meetings at GenP revealed that the regularity in contact depends on the individual's current status.

*When I went to CAP I met X regular, but now at GenP it's like, the worse you feel the more meetings you get, and the better you feel the less meetings you get, it's like more irregularly. (young adult)*

In cases when the transition process worked well, relatives and young adults felt that they had good luck. They were lucky receiving a medication that worked and they felt lucky getting a therapist who ensured a successful transition. From this point of view the outcome of the transition was a matter of chance instead of a well-planned process.

*It has gone quickly and smoothly, it was probably what I needed, I have avoided a lot of hassle, I was lucky I guess. (young adult)*

Society's and even professionals' attitudes toward mental illness contributed to a feeling of being omitted. Relatives described that it is not possible to talk about mental illness in the same way as about a physical illness. Relatives gave the example that nobody tries to hide taking medication for a physical illness, but when it comes to mental illness, it is shameful. They also felt that mental healthcare at GenP was not prioritized.

#### 3.4.2. Insecurity That Needs to Be Relieved

When relations at CAP were closed before summer vacation and new relations at GenP were not yet established, a caring gap arose, which led to insecurity and increased suffering. Both young adults and relatives described that the uncertainty about whom they would meet and when it would happen caused worries, anxiety, increased medication, and fears that ongoing medication would run out before they got an appointment. Relatives also worried that their young adults would decide not to continue care if professionals at GenP made the young adults responsible for making contact. During these circumstances of insecurity, relatives describe that they had great responsibility in supporting the young adults.

*We had to go several times with her and increase the medicine, she has felt really ill…even if she's talking to me but it's not the same thing…I'm not a therapist. (relative)*

Young adults could understand that professionals needed vacation, but they said that it was not optimal for them to make the closure during summertime because it became a long, uncertain waiting time for them.

*I would have been told more in time and then they should have fixed so I could meet someone during summer, a meeting with a doctor or anything…I think it sucks because I do not know how they think, if they think, yeah but now it's holiday, but it's not working so for all. (young adult)*

To bridge the caring gap, young adults and relatives asked for more cooperation between professionals at CAP and GenP and more flexibility on the appropriate time for transfer. They thought transition planning should be based on individual needs instead of professionals' working schedules so the young adults would not suffer because of a caring gap that could arise.

## 4. Discussion

The aim of this study was to explore expectations and experiences of transition from CAP to GenP. The results show that young adults were undergoing multiple simultaneous transitions. The transitions were complex for the participants because they had to adapt to new situations related to both developmental and situational changes [22–24]. According to Bridges [25], transitions are not only about the changes but also about the inner reorientation and self-redefinition a person goes through to incorporate the changes. Generally, the transition from childhood to adulthood is not a linear process since it includes a back and forth between independence and dependence and cannot be enforced pursuant a timetable [26]. For relatives it is also a balance between providing support and promoting independence [4] and it is important to support the relatives during the process of letting go [27]. According to McClure [28], adolescents in transition between childhood and adulthood have special needs that professionals have to understand and take into account. It is also important to take a family perspective as young adults are affected by their family and in turn also affect their family. Professionals have to consider that young adults undergo a process of emancipation from their family.

The young adults in this study had to adapt to a care culture with individual perspectives instead of a family perspective, although they were still in need of support to manage the transition from CAP to GenP. When young adults undergo multiple transitions, professionals must focus on both the situational transition to the new care unit and the developmental transition from childhood to adulthood [7, 29]. Meleis et al. [1] further stated that professionals need to consider the patterns of all significant transitions an individual or a family experiences, which include either a single transition or multiple transitions, and in the case of multiple transitions, either sequential or simultaneous. In this case, when young adults are transferred from CAP to GenP at the age of 18th they undergo multiple simultaneous transitions.

In this study, the young adults and the relatives described that they felt safe and secure at CAP because of close caring relations. Upon transfer to GenP, they had to leave these relations behind and establish new caring relationships at GenP. To promote young adults' willingness to disclose their mental health concerns and create relationships, they need a “space” in which a trusting relation might be built, and informal ways of keeping in touch can promote relations building [30]. In this study the young adults expressed that it takes time to establish trustful relationships and they expressed a need for continuity and confidence that professionals have skills to meet their current needs. It is, therefore, important to pay attention to young adult's narratives, in order to create trusting relationships in healthcare [31].

The results show further that young adults and relatives felt left out and had to rely on their own personal qualities. They described a lack of support and information about the future at GenP which increased the suffering and the risk of falling into the caring gap. Swift et al. [15] also describe that young adults were reliant on family support to take responsibility for their care and experience a feeling of being left out during the transition. According to Leavey et al. [30], young adults with mental illness most likely seek help from relatives and friends, and furthermore Ciarrochi et al. [32] found that young adults who are most in need of help and poor at managing their emotions least likely seek help when needed. This shows the importance of a well-planned transition process, through cooperation between CAP, GenP, and the families, to facilitate the young adults' ability to establish a caring relation and encourage continuity of care [33]. Otherwise, the risk is obvious that young adults will fall into the caring gap and not receive care when needed.

### 4.1. Methodological Considerations

The strength of this study is its insider perspective, that is, the young adults' and relatives' expectations and experiences. From this perspective, the result offers important insights into the sometimes unknown world of young adults with mental illness. The limitation of the study is primarily related to the sampling procedure. The sample was small and despite repeated attempts to draw additional participants, no more participants responded. However, according to Corbin and Strauss [20], the researcher is sampling concepts and not persons in theoretical sampling, and as the result was consistent with other studies in the same topic, the trustworthiness strengths. The categories and concepts contained in this study and the relationships between these categories are sufficiently abstracted to allow comparison to other clinical populations and other contexts, thereby enhancing the modifiability of the theoretical understanding. The results can be regarded as a synthesis of both research findings from this study and earlier research and knowledge in the field of transition. To reach a theory concerning transition from CAP to GenP, we need more empiric knowledge and theory embedded in nursing practice. Our results described in the core category can thereby be seen as a hypothesis that should be further explored to generate a theory of transition from CAP to GenP for young adults, contributing to theory development in nursing.

## 5. Conclusions

Based on these results, a person-centred transition planning is recommended to facilitate the transition from CAP to GenP. The framework for person-centred nursing comprises professionals' competence and interpersonal skills, the care environment, person-centred processes, and outcomes [34]. To decrease the risk of disruption in care and make the process person-centred, professionals should plan the transition through cooperation between CAP and GenP with the young adults and relatives participating. The professionals at both CAP and GenP also need to be aware of the simultaneous transitions young adults undergo and take into account their individual needs and maturity. Furthermore, it is important in person-centred nursing to pay attention to the care environment which includes system that facilitates effective relationship between professionals and a supportive organizational system [34]. In the context of CAP and GenP the gap between the different organizations and care cultures needs to be bridged. To achieve that, it could be an advantage to be more flexible in time for transfer. To facilitate young adults possibility to establish a caring relationship at GenP, parallel working during the transition process could be a benefit. With these interventions the young adults and the relatives will be prepared for the changes and they can manage the transition without a feeling of insecurity and uncertainty.

## Figures and Tables

**Table 1:** Overview of the analysis process.

Category	Subcategory	Interview text
Being of age but not mature	Still in need of support	I wanted company (relative) the first times we met because I didn't know the person, I didn't know what I should sit there and say (young adult)
		She is 19 but in many ways she's 13-14 years old and she is most probably not alone (relative)
	Being close yet letting go	It may be the hardest thing, you have been very close the whole life, but I have yet decided that I have to trust them…even if it feels like I'm not that important any more (relative)
		She should be involved as much as possible I think, but later on I can go there by myself…I want to decide if she should follow me to the meeting or not (young adult)

Walking out of security and into uncertainty	Achieving closure and starting again	I have vent to her for two three years and just started to work and so you have to start again with a new one and get to know and it will take some time…now I have start all over again from the beginning (young adult)
		It is important that the kids don't feel like they are thrown away…maybe you can meet two or three times depending on the situation (relatives)
	Leaving secure relations behind	I don't know, maybe because I met her so often and she helped me start talking…yeah we connected simply…some persons you like and some you don't and you must meet them to know if you will connect (young adult)
		I think we hit it off pretty well…she got to know me pretty well better than I knew myself (young adult)

Feeling omitted and handling concerns	Left to their fate	I don't like it, it sucks but I have no choice I just have to accept it (young adult)
		I must expect that they know what they do, that they have knowledge about different disabilities but I'm not sure (relative)
	Insecurity that needs to be relieved	The transfer shall take place in another way so it doesn't become an end and a gap (relative)
		Lack of knowledge about who when or anything, missing the security that disappeared, it has been really tough (relative)
